# Dyads of GGC and GCC form hotspot colonies that coincide with the evolution of human and other great apes

**DOI:** 10.1186/s12863-024-01207-z

**Published:** 2024-02-21

**Authors:** M. Arabfard, N. Tajeddin, S. Alizadeh, M. Salesi, H. Bayat, H. R. Khorram Khorshid, S. Khamse, A. Delbari, M. Ohadi

**Affiliations:** 1 Chemical Injuries Research Center, Systems Biology and Poisonings Institute, Baqiyatallah University of Medical Sciences, Tehran, Iran; 2 Iranian Research Center on Aging, University of Social Welfare and Rehabilitation Sciences, Tehran, Iran; 3 Department of Biology, Central Tehran Branch, Islamic Azad University, Tehran, Iran; 4 Research Center for Prevention of Oral and Dental Diseases, Baqiyatallah University of Medical Sciences, Tehran, Iran; 5 Personalized Medicine and Genometabolomics Research Center, Hope Generation Foundation, Tehran, Iran

**Keywords:** Human, Great ape, (GGC)2, (GCC)2, Colony, Recombination hotspot, Evolution

## Abstract

**Background:**

GGC and GCC short tandem repeats (STRs) are of various evolutionary, biological, and pathological implications. However, the fundamental two-repeats (dyads) of these STRs are widely unexplored.

**Results:**

On a genome-wide scale, we mapped (GGC)2 and (GCC)2 dyads in human, and found monumental colonies (distance between each dyad < 500 bp) of extraordinary density, and in some instances periodicity. The largest (GCC)2 and (GGC)2 colonies were intergenic, homogeneous, and human-specific, consisting of 219 (GCC)2 on chromosome 2 (probability < 1.545E-219) and 70 (GGC)2 on chromosome 9 (probability = 1.809E-148). We also found that several colonies were shared in other great apes, and directionally increased in density and complexity in human, such as a colony of 99 (GCC)2 on chromosome 20, that specifically expanded in great apes, and reached maximum complexity in human (probability 1.545E-220). Numerous other colonies of evolutionary relevance in human were detected in other largely overlooked regions of the genome, such as chromosome Y and pseudogenes. Several of the genes containing or nearest to those colonies were divergently expressed in human.

**Conclusion:**

In conclusion, (GCC)2 and (GGC)2 form unprecedented genomic colonies that coincide with the evolution of human and other great apes. The extent of the genomic rearrangements leading to those colonies support overlooked recombination hotspots, shared across great apes. The identified colonies deserve to be studied in mechanistic, evolutionary, and functional platforms.

## Introduction

Short tandem repeats (STRs), also referred to as microsatellites or simple sequence repeats, play a significant role in evolution and disease [1–13]. GGC and GCC repeats are particularly linked to natural selection due to several reasons, including enrichment in genic region [14, 15], predisposition to mutations [1, 2, 16–18], frequent order-specificity of these STRs, expanded GGC and GCC repeats in various neurodevelopmental, neurodegenerative, and movement disorders [19, 20], and lastly, indications of unambiguous genotypes at certain GGC and GCC STRs in late-onset neurocognitive disorders, such as Alzheimer's disease and cerebrovascular dementia [1–3].

The fundamental two-repeats (dyads) of STRs are largely overlooked in genetic and genomic studies. Based on the biological, evolutionary, and pathological implications of GGC and GCC STRs, in a pilot study, we chose to investigate dyads of these STRs, i.e., (GGC)2 and (GCC)2. We mapped the (GGC)2 and (GCC)2 dyads across the human genome, and identified genomic colonies of these dyads, of exceeding significance, based on Poisson probability. Several of the largest colonies that were further studied in additional species, were found to be specific to the human species, or while shared with other great apes, were at maximum complexity in human. Our findings unveil dyad colonies of evolutionary relevance and overlooked shared recombination hotspot loci across human and other great apes.

## Methods

### Genomic (GGC)2 and (GCC)2 extraction

The UCSC genome browser (https://hgdownload.soe.ucsc.edu) was utilized to download the most recent version of the human genome assembly, GRCh38.p14. To investigate the abundance of the (GGC)2 and (GCC)2 dyads throughout the entire genome, a Java software package was developed. The software package can be found at the following GitHub repository: https://github.com/arabfard/Java_STR_Finder. Our approach involved searching for annotations of (GGC)2 and (GCC)2 on both the forward and reverse strands of the genome. The software extracted a list of (GGC)2 and (GCC)2 dyads, along with their respective genomic locations. To validate the accuracy of the tool, a random selection of these dyads was manually inspected across the genome.

### Details of extraction algorithm

A written program was used to identify (GGC)2 and (GCC)2 in the human genome. The program followed a specific method, starting from the first nucleotide and moving across the genome nucleotide by nucleotide. In the first stage, the program examined a window frame of size 6 (2 * 3), where 2 represented the number of tandem repetitions and 3 represented the length of the GGC or GCC core. If the initial half of the sequence within the window did not match the second half, the program moved one nucleotide forward. If the nucleotides were equal, the program continued examining them until it located all identical continuous nucleotides matching the core. The final chosen sequence, represented as (GGC)2 or (GCC)2 with a core length of 3 and repetition of 2, was considered a new dyad. To find additional dyads, the entire process was repeated starting from the end of the preceding dyad.

To validate the obtained data, the final list of information was manually evaluated using Ensembl genome browser 109 (https://asia.ensembl.org/index.html). The identified locations of (GGC)2 and (GCC)2 dyads were then manually determined using the Ensembl database 109. The algorithm's output was classified in an Excel file, and for each dyad, the start and end points on the genome were determined (with the sequence address provided in another column). The detailed data can be accessed at the URL: https://figshare.com/articles/dataset/_GGC_2_and_GCC_2/22178102. To identify the colonies, a method was employed where the start and end points of the next dyad were calculated. If the difference between these points was < 500 bp, they were considered candidate colonies. The colonies containing (GGC)2 and/or (GCC)2 dyads were then highlighted, and the total number of colonies was determined. The detailed information about these colonies can be found at the URL: https://figshare.com/articles/dataset/_GGC_2_and_GCC_2/22178102.

### Screening selected colonies of (GGC)2 and (GCC)2 in human and other species

The Ensembl Genome Browser 109 (https://asia.ensembl.org/index.html) BLASTN program was utilized to examine several of the largest colonies in several species of primate and rodent orders.

### Statistical analysis

Given the assumption that the number of (GGC)2 and (GCC)2 elements in the entire genome is known, their distribution can be modeled as a Poisson process. The number of these elements within a specific interval follows a Poisson distribution with an average proportional to the length of the interval.

In this study, considering the wide range of detected colony locations, it was assumed that these dyads are distributed relatively evenly across the genome. Consequently, the probability of colony occurrence was calculated using the Poisson density function with the following parameter:

$$\uplambda =\frac{\left(26\ \text{kb}\right)\: *\ {\text{genome}}-\text{wide dyads of}({\text{GGC}})2\ \text{and }\left({\text{GCC}}\right)\!\,2}{\text{genome size}\ (\simeq\ 3{\text{gb}})}$$

## Results

### (GGC)2 and (GCC)2 dyads formed colonies across the human genome

**Fig. 1:**
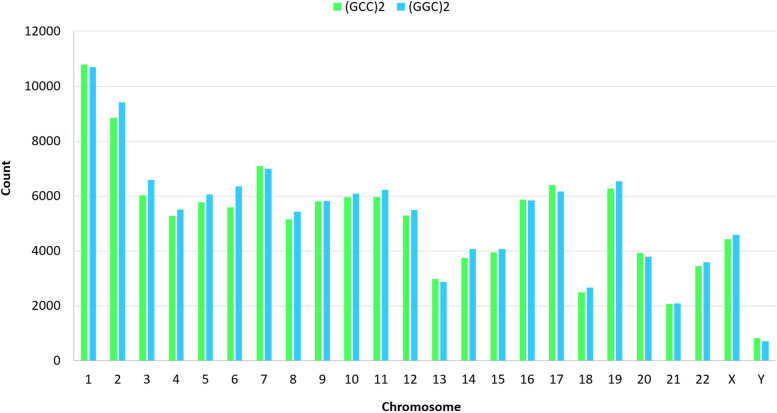
Chromosome by chromosome distribution of (GGC)2 and (GCC)2 in human

**Fig. 2:**
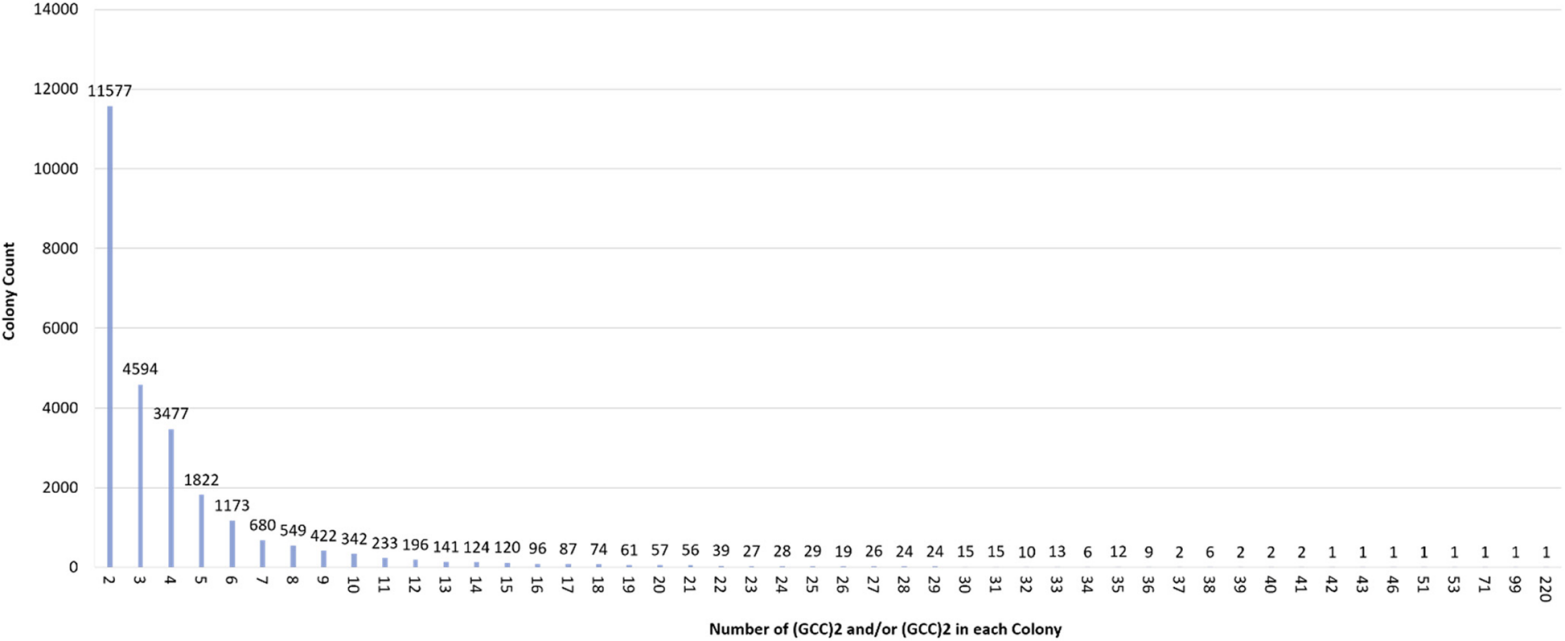
Genome-wide abundance of various colony sizes of (GGC)2 and (GCC)2 in human

According to the dataset available at  https://figshare.com/articles/dataset/_GGC_2_and_GCC_2/22178102, a total of 127,770 occurrences of (GGC)2 and 124,023 occurrences of (GCC)2 were identified throughout the human genome. Among those, 26,199 instances formed colonies, i.e., the dyads were located within a distance of < 500 bp from each other (Figs. 1 and 2).

The distribution of (GGC)2 and (GCC)2 was found to be non-proportional to the length of several chromosomes (*p* < 0.000). This observation indicates that the occurrence of these dyads is not random. Additionally, various size colonies were associated with highly significant occurrence of these colonies, as indicated by statistical analysis (Table 1).

**Table 1:** Poisson probability of various colony sizes

Colony Size	Probability	Colony Size	Probability
2	0.020541568	26	3.59193E-44
3	0.001554709	27	3.02065E-46
4	8.82523E-05	28	2.44951E-48
5	4.00768E-06	29	1.91787E-50
6	1.51663E-07	30	1.45156E-52
7	4.91946E-09	31	1.06319E-54
8	1.39625E-10	32	7.5439E-57
9	3.52256E-12	33	5.19062E-59
10	7.99825E-14	34	3.46639E-61
11	1.65097E-15	35	2.24877E-63
12	3.12388E-17	36	1.41834E-65
13	5.45617E-19	37	8.70392E-68
14	8.84906E-21	38	5.20078E-70
15	1.3395E-22	39	3.02789E-72
16	1.9009E-24	40	1.71877E-74
17	2.53891E-26	41	9.51854E-77
18	3.20266E-28	42	5.14586E-79
19	3.82732E-30	43	2.71723E-81
20	4.34512E-32	46	3.49232E-88
21	4.69807E-34	51	7.4769E-100
22	4.84879E-36	53	1.3987E-104
23	4.78677E-38	71	1.809E-148
24	4.52864E-40	99	1.545E-220
25	4.11305E-42	219	0

### The top largest (GCC)2 and (GGC)2 colonies in human

#### (GCC)2 colonies

**Fig. 3:**
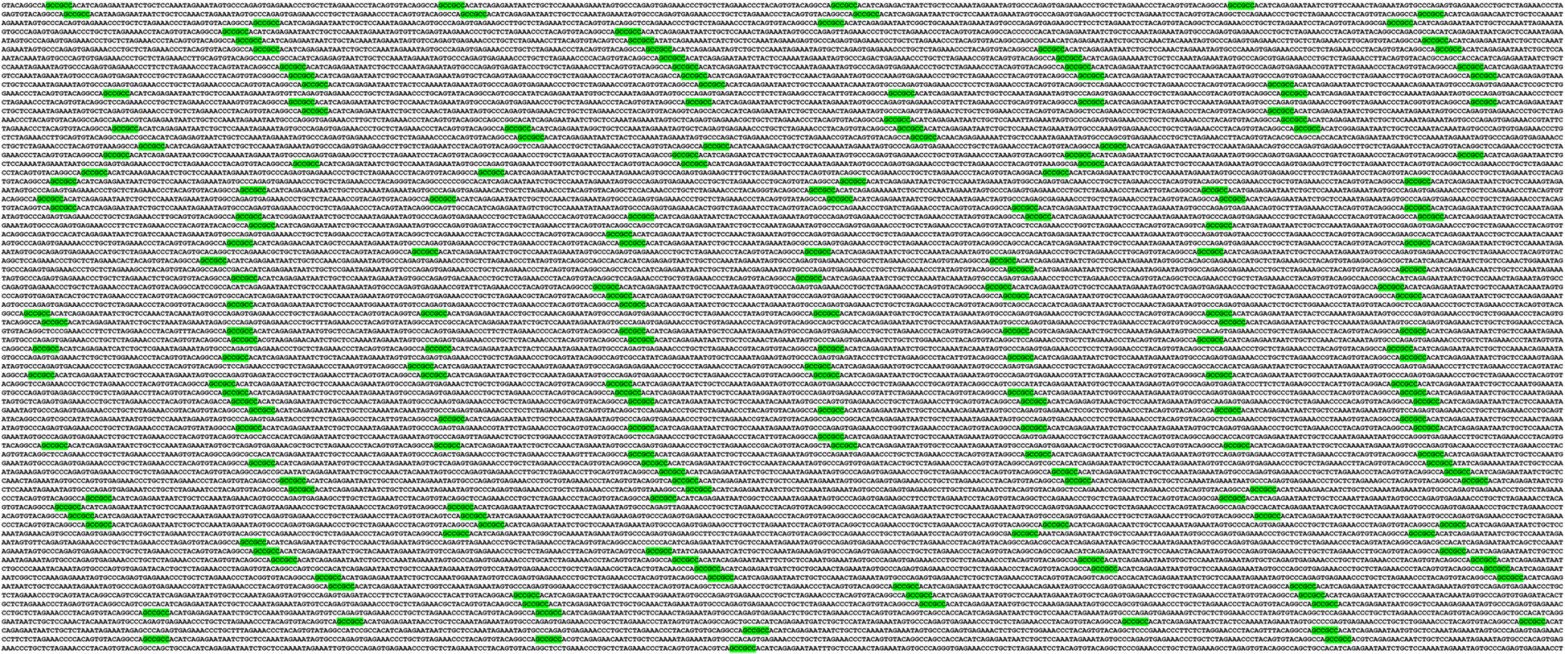
The largest (GCC)2 colony in human (C219). This gigantic, intergenic, and homogeneous colony consists of 219 (GCC)2, and the nearest gene to this colony is *COPS7B*, which is nearly 14 kb upstream of this colony. This colony is human-specific i.e., trace of (GCC)2 was non-existent across other species. (GCC)2 are green-highlighted

The largest (GCC)2 colony, comprising 219 (GCC)2 dyads, i.e., (C219), was identified on chromosome 2, in an intergenic region (Table 2, Fig. 3). Notably, this colony was found to be specific to human.

**Table 2:** Several of the top largest (GCC)2 and (GGC)2 colonies across human genome

Colony Formula	Chr. No	Location	Transcript ID	Biotype
[(GCC)2]219	2	Intergenic^a^ (14 kb downstream of *COPS7B*)	ENST00000350033.8	
[(GCC)2]99	20	Intergenic (5 kb downstream of *CDH4*)	ENST00000611855.4	
[(GGC)2]70 (GCC)2	9	Intergenic (16kb upstream of *WDR5*)	ENST00000358625.4	
[(GCC)2]51	16	*RAB40C* (Intron 1)	ENST00000248139.8	Protein coding
[(GGC)2]41	4	Intergenic (21 kb downstream of *TRAPPC11*)	ENST00000334690.11	
[(GGC)2]38 (GCC)2	10	*ABCC2* (Intron 25)	ENST00000647814.1	Protein coding
[(GGC)2]38	19	Intergenic (14 kb downstream of *CYP2B7P*)	ENST00000599198.5	
[(GCC)2]36	X, Y	*IL3RA* (Intron 9 pseudo autosomal region)	ENST00000331035.10	Protein coding
[(GGC)2]35	X	*KDM6A* (Intron 8)	ENST00000611820.5	Protein coding
[(GGC)2]33	4	Intergenic (109 kb downstream of *COPS4*)	ENST00000264389.7	
[(GCC)2]32	16	Intergenic (118 kb downstream of *SETD1A*)	ENST00000262519.14	
[(GCC)2]30	18	*ANKRD20A5P* (Intron 15)	ENST00000431648.8	Transcribed unprocessed pseudogene
[(GCC)2]20 (GGC)2	17	Intergenic (160 kb upstream of *COPS3*)	ENST00000268717.10	
[(GGC)2]11 [(GCC)2]5	17	*KANSL1* (promoter/5′ UTR)	ENST00000262419.10	Protein coding
[(GGC)2]16	Y	*TTTY10* (Intron 1)	ENST00000661812.1	lncRNA
[(GCC)2]8 [(GGC)2]8	11	Intergenic (229 kb downstream of *MACROD1*)	ENST00000255681.7	
[(GCC)2]11	Y	*XGY1* (Intron 6)	ENST00000381172.3	Unprocessed pseudogene
[(GCC)2]6 [(GGC)2]4	3	Intergenic (197kb downstream of *WDR82*)	ENST00000296490.8	

^a^For the intergenic colonies, the nearest gene to those colonies is annotated

**Fig. 4:**
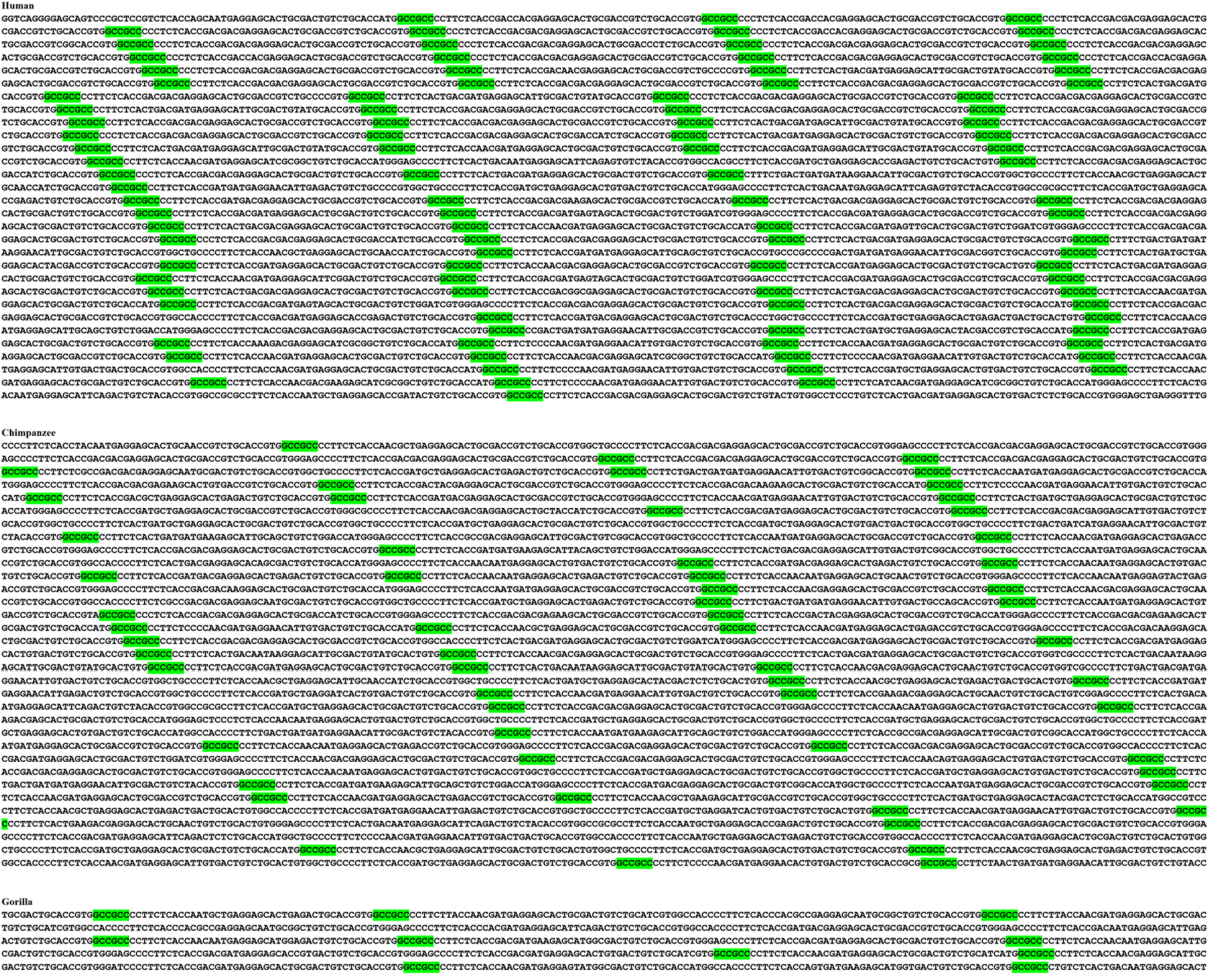
Directional incremented complexity and density of an intergenic homogeneous (GCC)2 colony (C99) in human versus other species. This colony was located 5 kb downstream of *CDH4*, and was specific to great apes. (GCC)2 are green-highlighted. This colony signifies a novel recombination hotspot shared between human and other great apes

The second largest colony consisted of 99 (GCC)2 dyads, (C99), and was located 5 kb downstream of the cadherin 4 (CDH4) gene. Interestingly, this homogeneous colony was specific to great apes. Furthermore, our analysis revealed a directional incremented complexity and density of this colony in human, compared to other great apes (Fig. 4).

**Fig. 5:**
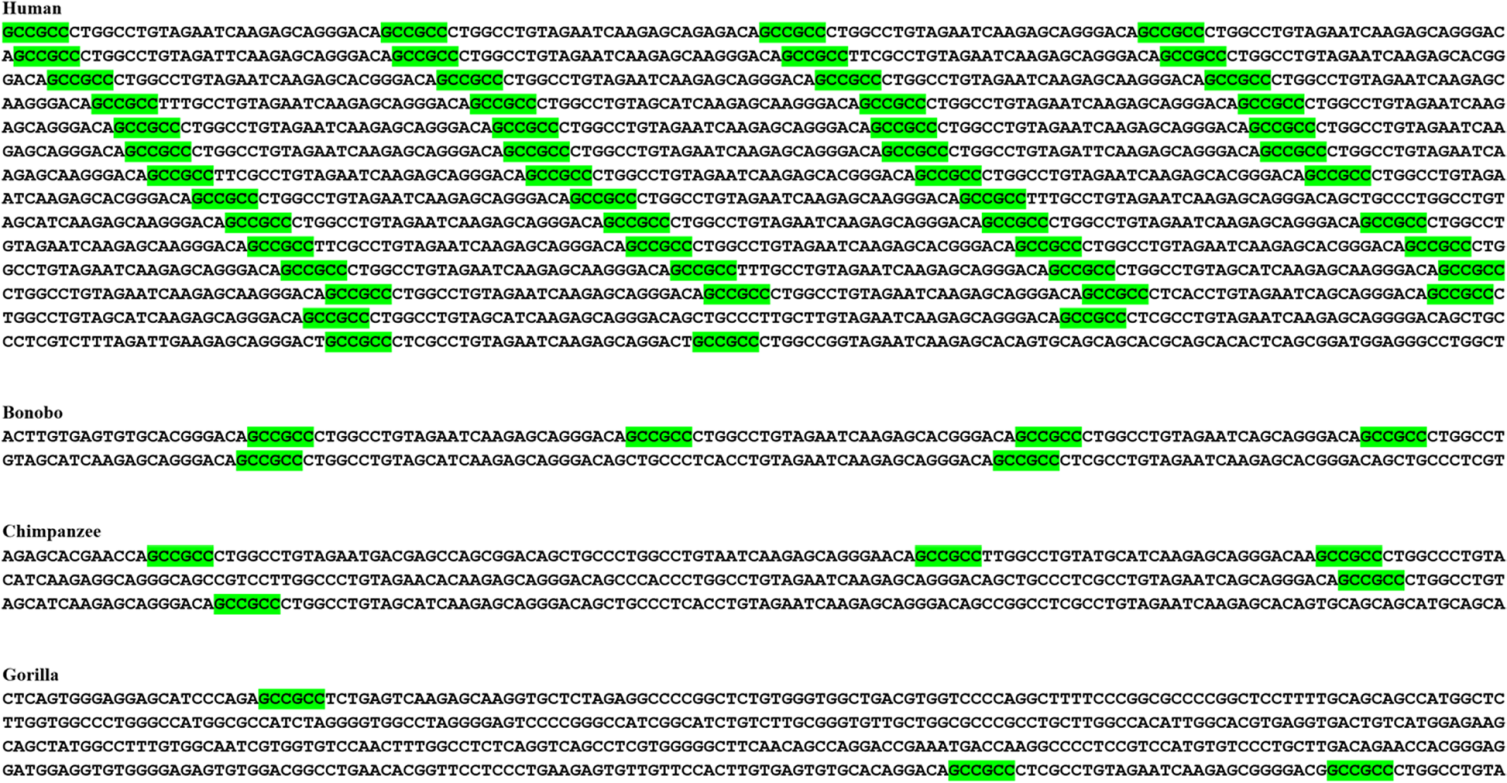
Directional incremented complexity and density of an intragenic (GCC)2 colony in human (C51). This homogeneous colony was within *RAB40C*, specific to great apes, and reached maximum complexity in human. This colony may unfold a novel recombination hotspot shared by great apes. (GCC)2 are green-highlighted

Another example of a directional trend observed in humans compared to other species was the RAB40C colony (C51) (Fig. 5). This colony was specific to great apes, and exhibited a significant increase in complexity in humans, reaching its maximum complexity in human (Fig. 5). This finding suggests that the RAB40C colony has undergone evolutionary changes, potentially contributing to the unique characteristics of the human species.

#### (GGC)2 colonies

**Fig. 6:**
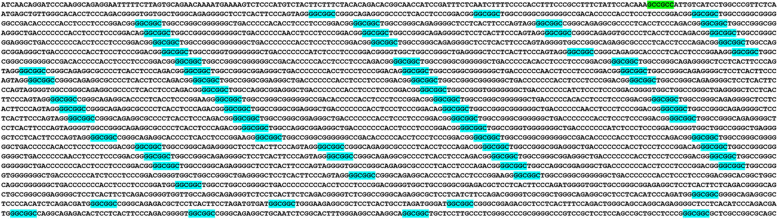
The largest homogeneous (GGC)2 colony in human (C70). This colony is human-specific and located 16 kb upstream of the *WDR5* gene. (GGC)2 are blue-highlighted. (GCC)2 is green-highlighted

The largest (GGC)2 colony, C71, was located 16 kb upstream of the WDR5 gene, and was specific to human. This colony exhibited a predominantly homogeneous composition (Fig. 6).

**Fig. 7:**
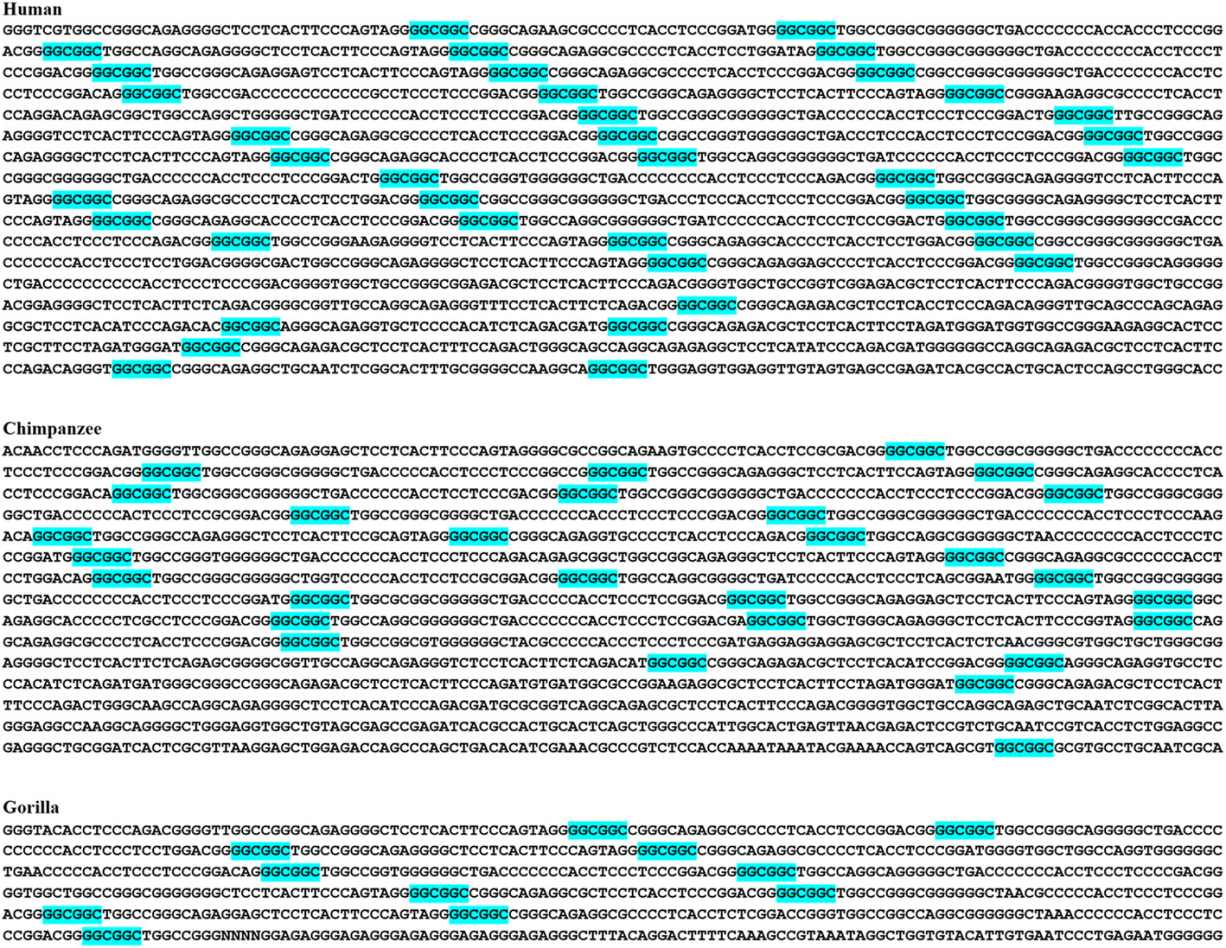
Example of a (GGC)2 colony with directional incremented complexity in human (C38). The colony is 14 kb downstream of *CYP2B7P*, specific to great apes, and maximally complex in human. This colony may unfold a common recombination hotspot in great apes

Additionally, directional trends were observed for (GGC)2 colonies, when comparing humans to other species. For instance, the [(GGC)2]38 colony (Table 2) was specific to great apes. This colony reached its maximum complexity and density in the human genome (Fig. 7).

### Chromosomes X and Y harbor numerous colonies of (GGC)2 and (GCC)2

**Fig. 8:**
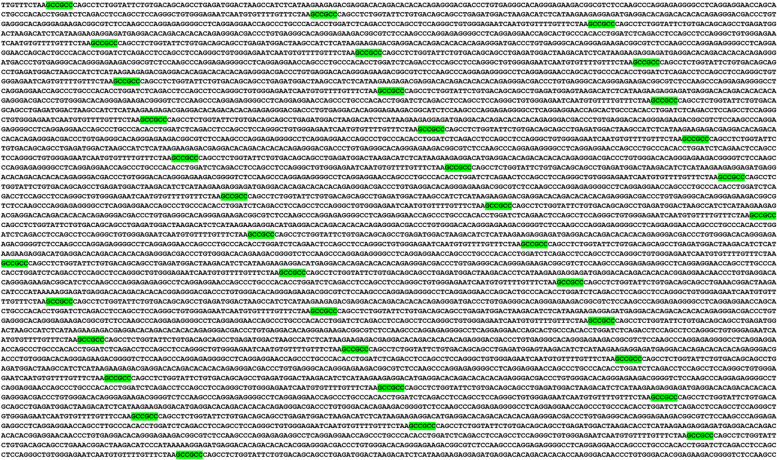
Example of a human-specific pseudoautosomal colony (C36). This homogeneous colony is located in *IL3RA*. (GCC)2 are green-highlighted. This colony contains prime instance of LTR tandemization in the human genome

Several colonies of (GGC)2 and (GCC)2 dyads were detected on chromosomes X and Y (Table 2). For example, C36 was located in the pseudoautosomal regions of these chromosomes, was human-specific, and located in the IL3RA gene (Fig. 8).

In several instances, not only were the colonies human-specific, but the genes containing those colonies were also specific to the human genome, such as C17 in the long non-coding RNA (lncRNA) gene TTTY10, (Table 2).

Colonies of (GGC)2 and (GCC)2 dyads were detected in pseudogenes as well. One such example is C11, in the XGY1 pseudogene (Table 2). This particular colony was specific to great apes, and reached its maximum size in the human genome. This observation underscores the importance of considering pseudogenes in the context of CG-rich dyads, and their potential impact on genome dynamics.

## Discussion

The significance of STRs in biological, evolutionary, and pathological contexts is an expanding area of research. However, the fundamental and most basic repeats of these elements, such as (GGC)2 and (GCC)2, are largely unexplored. In this study, we aimed to address this gap, which resulted in the identification and characterization of unprecedented genomic colonies, formed by these dyads. Our findings revealed numerous colonies that were specific to humans or exhibited directional incremented complexity when comparing humans to other species. These observations, combined with the statistically significant occurrence of these colonies, lead us to propose that these (GGC)2 and (GCC)2 colonies may play a role in the evolution of the human species. By shedding light on the overlooked basic repeats of STRs and their genomic coloniza tion, our study provides new insights into the potential importance of these elements in the evolutionary processes that have shaped the human genome.

The genomic rearrangements in the identified colonies are remarkable in terms of their frequency within the genomic lengths that they occurred. These colonies do not conform to the conventional description of segmental duplications, as the shortest reported human segmental duplications and copy number variations involve genomic DNA lengths of at least 10 kilobases (kb) in humans [21–24]. The likely explanation for the occurrence of these colonies is recombination, involving the dyads and the flanking sequences around each dyad. In other words, the identified colonies can be considered recombination hotspots. Previous studies comparing fine-scale recombination rates in humans and chimpanzees have reported rapid evolution of local recombination patterns, which are often not conserved between the two species [25]. However, if we assume that the identified colonies are at least partially formed by recombination, it suggests that common recombination hotspots at the same genomic locus between the two species are not as rare as previously reported. For example, the colonies C99, C51, and C38 are likely to be shared recombination hotspots in great apes, albeit with higher complexity in humans. These examples demonstrate prime instances, where the directional incremented density and complexity of repeats at specific loci in the genome coincide with human evolution. Another example includes a CT-repeat complex in the *PAXBP1* core promoter and 5' untranslated region, which exhibits maximal complexity in human compared to other species (OMIM: 617,621) [26]. These findings underscore the potential role of recombination hotspots in shaping genomic rearrangements and their association with the evolutionary changes observed in the human genome. Based on the fact that the main elements, in common, across the colonies are the dyads, it is likely that the main reason for the rearrangement hotspots in the identified colonies is the dyads, rather than their flanking sequences.

**Fig. 9:**
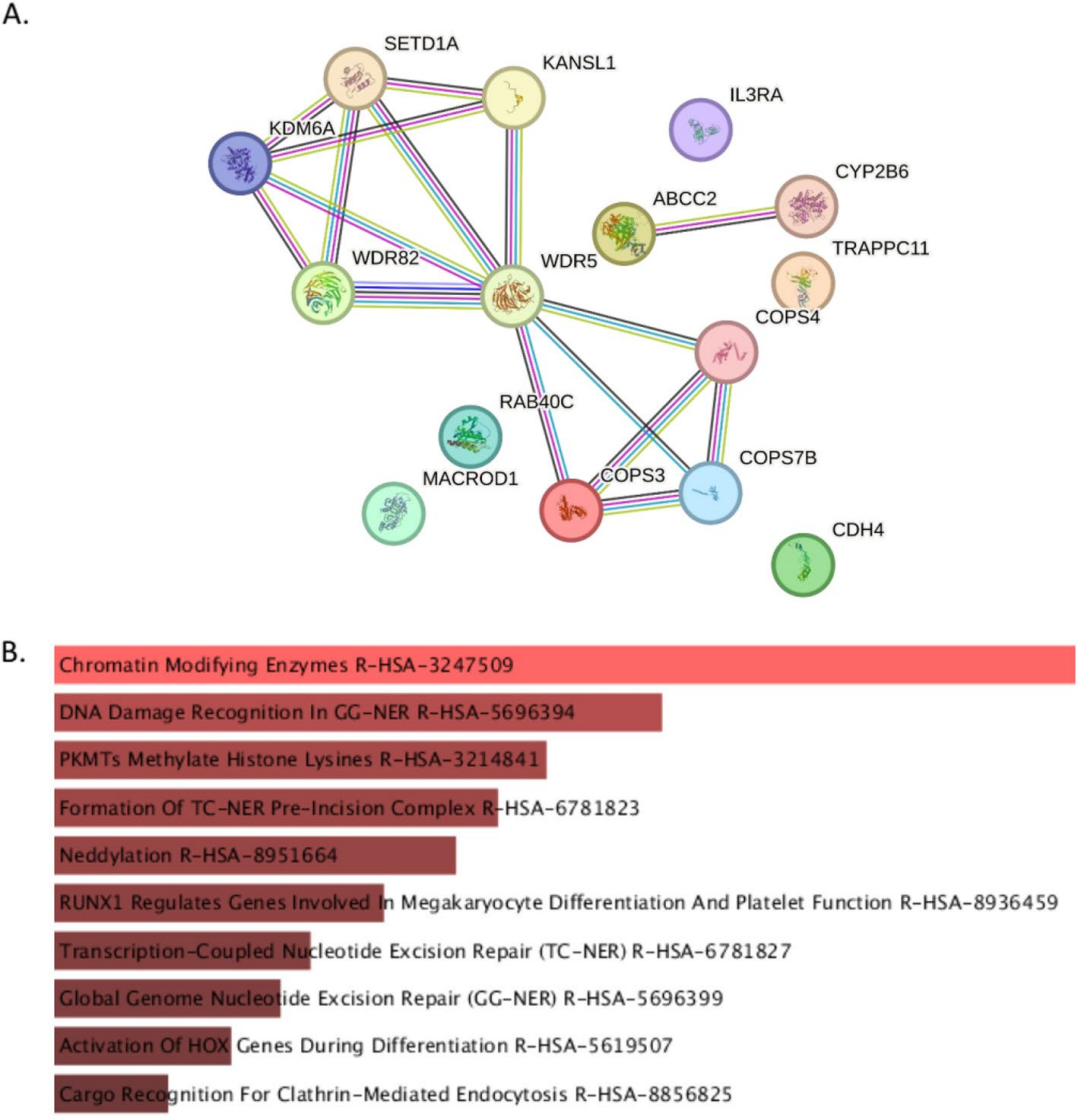
Interactions and biological role of the genes containing (or nearest to) the largest colonies. **A** Protein–protein interaction network, **B** Biological pathway enrichment analysis

Several of the genes, which contained (or were nearest to) the top largest colonies (Table 2) interacted closely at the protein level (https://string-db.org) (Fig. 9A), and were enriched in chromatin remodeling and histone modification pathways (Fig. 9B).

For example, C219 and C71 were intergenic, and the nearest genes to those colonies were *COPS7B* and *WDR5*, respectively, which directly interact at the protein level. Intergenic distance and genome architecture are known to be non-random and influenced by regulatory information present in noncoding DNA [27]. The expansion of the non-coding genome and its regulatory potential have been implicated in vertebrate neuronal diversity. It is not surprising, therefore, that the largest colonies, which are mainly human-specific or more complex in humans compared to other species, are associated with genes that exhibit divergent expression in the human brain [28]. This information is supported by research available at the Assembly resource (https://www.ncbi.nlm.nih.gov/IEB/Research/Acembly/), [29]. A subset of the (GCC)2 and (GGC)2 colonies were found deep within large introns. It is noteworthy that for certain genes, the regulatory sequences of importance are not located in the promoters, but rather within introns [30–32].

Remarkably, in C36, we detected tandem long terminal repeats (LTRs) (https://genome.ucsc.edu/). C36 is a pseudoautosomal gene, located in the immune gene, IL3RA. To our knowledge, this colony is prime example of LTR tandemization in the human genome. Similar to the other colonies, the mechanism of tandemization in this colony may be linked to the dyads. It should be noted that instances of retrotransposon tandemization (such as the LTRs in C36) in human are rare. An exceptional instance of short interspersed nuclear element (SINE) tandemization has been recorded in connection with (GAA)n (for a review see [33]).

Some of the identified colonies were found in close proximity to long non-coding RNAs (lncRNAs). Although the exact targets of many lncRNAs are not fully understood, they have gained significant attention due to their versatile roles in fine-tuning various signaling pathways [34]. Another category of colonies was found within pseudogenes. Some of those colonies were specific to great apes, and exhibited directional trend of increased complexity and size in human. Pseudogenes, once considered nonfunctional gene remnants, are abundant in the human genome. However, recent observations suggest that pseudogenes play a role in regulating gene expression both transcriptionally and post-transcriptionally in human cells. Pseudogenes are transcribed on both strands and are significant drivers of gene regulation, with implications for health and diseases [35–37].

It should be noted that this is a pilot study, which unveils the potential significance of trinucleotide dyads in shaping part of the recombination landscape in the human genome, and challenges the long-lasting hypothesis that human and closely related species do not share recombination hotspots. Numerous other trinucleotide dyads and additional species are yet to be studied in this context, to obtain a more resolved perspective of the role of trinucleotide dyads in recombination, speciation, and evolution.

## Conclusion

In conclusion, our findings unveil a genomic phenomenon, characterized by the formation of large colonies of (GGC)2 and (GCC)2 dyads of exceeding statistical significance throughout the human genome. These colonies exhibit unprecedented frequency and, in some instances, periodicity of genomic rearrangements, signifying recombination hotspots. Some of the identified colonies that were further studied in additional species, were specific to human, or were shared with other great apes, albeit of directional increased complexity in human. Future studies are warranted to unveil the mechanisms leading to the emergence of those colonies and their biological implications.

## Data Availability

All raw data are available in at the following link: https://figshare.com/articles/dataset/_GGC_2_and_GCC_2/22178102.

## Abbreviations

C: Colony
kb: Kilobase
Gb: Gigabase
LTR: Long terminal repeat
STR: Short tandem repeat

## Glossary

Colony: Consecutive (GGC)2 and/or (GCC)2 that were <500 bp apart on the genomic DNA
Dyad: (GCC)2 or (GCC)2
Homogeneous: Applied to colonies that primarily consisted of a single dyad type
Human-specific: Indicates the absence of (GGC)2 or (GCC)2 traces in other species
